# Digoxin, an Overlooked Agonist of RORγ/RORγT

**DOI:** 10.3389/fphar.2018.01460

**Published:** 2019-01-07

**Authors:** Kaja Karaś, Anna Sałkowska, Marta Sobalska-Kwapis, Aurelia Walczak-Drzewiecka, Dominik Strapagiel, Jarosław Dastych, Rafał A. Bachorz, Marcin Ratajewski

**Affiliations:** ^1^Laboratory of Epigenetics, Institute of Medical Biology, Polish Academy of Sciences, Lodz, Poland; ^2^Biobank Lab, Department of Molecular Biophysics, Faculty of Biology and Environmental Protection, University of Lodz, Lodz, Poland; ^3^Laboratory of Cellular Immunology, Institute of Medical Biology, Polish Academy of Sciences, Lodz, Poland; ^4^Laboratory of Molecular Modeling, Institute of Medical Biology, Polish Academy of Sciences, Lodz, Poland

**Keywords:** RORγ, RORC, Th17, agonist, digoxin, molecular docking

## Abstract

Digoxin was one of the first identified RORγT receptor inverse agonists inhibiting the differentiation of Th17 cells. However, this compound exhibits inhibitory activity at relatively high concentrations that mediate cytotoxic effects. We previously identified several cardenolides that are structurally similar to digoxin that were able to induce RORγ/RORγT-dependent transcription. These observations encouraged us to reanalyze the effects of digoxin on RORγ/RORγT-dependent transcription at low, noncytotoxic concentrations. Digoxin induced RORγ/RORγT-dependent transcription in HepG2 and Th17 cells. Furthermore, analysis of the transcriptomes of Th17 cells cultured in the presence of digoxin revealed the induction of the expression of numerous Th17-specific genes, including IL17A/F, IL21, IL22, IL23R, CCR4, and CCR6. Thus, our study, which includes data obtained from intact cells, indicates that digoxin, similar to other cardenolides, is a potent RORγ/RORγT receptor activator and that its structure may serve as a starting point for the design of dedicated molecules that can be used in the development of adoptive cell therapy (ACT).

## Introduction

The nuclear receptor (NR) superfamily consists of 48 ligand-activated transcription factors that are involved in a variety of physiological functions. Members of this superfamily have a typical domain structure (Burris et al., 2013). The first domain, the N-terminal A/B domain, is the most divergent among the NRs and is frequently posttranslationally modified. In many receptors, an AF-1 (activation function 1) region within the A/B domains is responsible for interactions with corepressors and coactivators, independent of ligand binding (Warnmark et al., 2003). The DNA binding domain (DBD, domain C) is the most conserved region of the nuclear receptors and is responsible for recognizing and binding response elements (REs) within the regulatory regions of target genes. Domain D, also called the hinge region, probably has a regulatory function that determines the interaction of the receptor with other proteins or mediates protein translocation (Horlein et al., 1995; Pissios et al., 2000; Haelens et al., 2007). The next domain, termed the ligand-binding domain (LBD, domain E), contains the AF-2 (activation function 2) region, which acts in a ligand-dependent manner (Jetten, 2009; Solt et al., 2010; Burris et al., 2013).

The involvement of nuclear receptors in the regulation of developmental and physiological processes, including those underlying many diseases, e.g., cancer and immunological and endocrine disorders, makes them interesting targets for drug development. A good example of such a drug development approach is the search for molecules inhibiting the transcriptional properties of the protein products (two isoforms) of the *RORC* gene (retinoic acid-related orphan receptor C): RORγ and RORγT. The two isoforms, which differ by only 21 amino acids in their N-terminal A/B domains, have different tissue distributions and probably have different functions. The longer isoform, RORγ, is broadly expressed (He et al., 1998) and regulates genes involved in the circadian cycle and metabolism (Kang et al., 2007; Jetten, 2009; Takeda et al., 2012) while the shorter isoform, RORγT, is exclusively expressed in Th17 cells, where it regulates their development and the expression of the signature interleukins IL17A and IL17F (Ivanov et al., 2006; Crome et al., 2009). Due to the involvement of Th17 in pathogenic processes underlying autoimmunological diseases, e.g., rheumatoid arthritis (Hirota et al., 2007), Graves’ disease (Zheng et al., 2013), and multiple sclerosis (Kebir et al., 2007), RORγT is perceived as a promising target in the development of new pharmaceuticals for the treatment of autoimmunological diseases by modulating the pathogenic activity of Th17. One of the first identified molecules affecting the function of RORγT was digoxin (Huh et al., 2011), which is a derivative of plants in the genus *Digitalis* that belongs to a group of compounds known as cardenolides. In a mouse model, it has been shown that treatment with high doses of digoxin has positive effects against experimental colitis (Xiao et al., 2014; Tani et al., 2017) and atherosclerosis (Shi et al., 2016) and attenuates acute cardiac allograft rejection (Wu et al., 2013).

Previously, in a screening study of two chemical libraries, we identified three cardenolides with activatory properties toward RORγ/RORγT, and digoxigenin, an aglycon of digoxin, was among them (Karaś et al., 2018). This prompted us to reevaluate the impact of digoxin on RORγ/RORγT nuclear receptors. We found that at nontoxic nanomolar concentrations, digoxin was able to induce RORγ-dependent transcription in HepG2 cells and RORγT-dependent expression in human Th17 cells. Thus, our results show, for the first time, that digoxin acts as an agonist activating human RORγ/RORγT.

## Materials and Methods

### Cell Culture

The HepG2 (human hepatocellular carcinoma) cell line was purchased from American Type Culture Collection (ATCC, Manassas, VA, United States) and cultured in Dulbecco’s Modified Eagle’s Medium (DMEM) with high (4.5 g/l) glucose completed with 10% fetal bovine serum (PAN Biotech GmbH, Aidenbach, Germany) at 37°C in an atmosphere of 5% CO_2_. The reporter cell line HepG2-RORγ stably transfected with the reporter plasmid (RORE)6-tk-Luc (Salkowska et al., 2017) containing six copies of RORE (5′-GGTAAGTAGGTCA-3′) (Medvedev et al., 1996), as described in our previous study (Karaś et al., 2018) was cultured, similarly, to the maternal HepG2 cells but with the presence of 50 μg/ml hygromycin B.

### Cell Viability

The cytotoxicity of digoxin in HepG2 cells was established with a neutral red uptake assay (Repetto et al., 2008). In detail, cells were plated into 96-well transparent plates at a density of 1.5 × 10^4^ cells per well. After overnight culturing, the cells were treated with increasing concentrations of digoxin for 24 h. Then, the medium was removed, and the cells were washed with a cold buffered saline solution. Neutral red was added (50 μg/ml) to the cells, and the plates were incubated for 3 h to allow neutral red penetration into the cells. After incubation, the neutral red solution was discarded, and the cells were washed with buffered saline solution. To extract the cell-bound dye, a solution containing 50% ethanol and 1% acetic acid was added. The absorbance of each sample was measured at 550 nm using a Sunrise microplate reader (Tecan, Männedorf, Switzerland). The viability of Th17 cells cultured for 5 days in the presence of different concentrations of digoxin was established with CellTiter-Glo^®^ Luminescent Cell Viability Assay (Promega, Madison, WI, United States) according to manufacturer’s instructions.

### Plasmid and Reagents

The construction of the human RORγ expression plasmid was described previously (Karaś et al., 2018). Expression vectors containing human RORγT and mouse Rory/Roryt cDNA and a control pCMV6-XL5 vector were purchased from OriGene Technologies (Rockville, MD, United States). The reporter vector pGL4.35[luc2P/9XGAL4UAS/Hygro] was purchased from Promega (Madison, WI, United States). The GAL4-DBD RORα and GAL4-DBD RORγ fusion constructs were described previously (Kumar et al., 2010) and were a kind gift from Prof. Patrick Griffin. Hygromycin B (P06-08020) was purchased from PAN Biotech GmbH. Digoxin (D6003, purity ≥ 95.0%) was purchased from Sigma-Aldrich (Saint Louis, MO, United States).

### Stable Transfection and Generation of the HepG2-pGL4.35 Reporter Cell Line

HepG2 cells were seeded into 6-well plates in DMEM medium and allowed to reach approximately 70% confluency. The cells were transfected with 2 μg of pGL4.35[luc2P/9XGAL4UAS/Hygro] reporter plasmid using Fugene HD (Promega). Forty-eight hours after transfection, cells were selected with 50 μg/ml hygromycin B (Sigma-Aldrich) for 4 weeks. Hygromycin B-resistant colonies were isolated, expanded and frozen in aliquots for subsequent experiments.

### Transient Transfection and Luciferase Assay

The RORγ-HepG2 cells were seeded into 96-well white plates at a density of 1.5 × 10^4^ cells per well, and 24 h later, they were treated with increasing concentrations of digoxin for another 24 h. Luciferase activity in the cell lysates was measured using an Infinite^®^ 200 PRO (Tecan, Männedorf, Switzerland) with d-luciferin substrate (Cayman Chemical, Ann Arbor, MI, United States). In experiments where the expression vectors were used, the RORγ-HepG2 and HepG2-pGL4.35 cells were also cotransfected with the pCMV-SEAP vector (a kind gift from Dr. S. Schlatter, Zurich), and the alkaline phosphatase control signal was measured spectrophotometrically at 405 nm as a transfection efficiency control.

### Naive CD4+ T Cell Isolation and Differentiation Into Th17 Cells

Peripheral blood mononuclear cells (PBMCs) were isolated from buffy coats obtained from healthy, anonymous donors by centrifugation through Ficoll. Buffy coats were bought as waste material from Regional Center for Blood Donation and Blood Treatment (Łódź, Poland). The naive CD4+ fraction was isolated using CD4 M-pluriBead^®^ anti-hu beads (pluriSelect Life Science, Leipzig, Germany). Th17 lymphocytes were obtained according to the protocol described by Wilson et al. (2007). Briefly, naive CD4+ cells were cultured under Th17-polarizing conditions (for 5 days) in Yssel’s medium containing human AB serum and the following cytokines: 50 ng/ml human IL-1β, 30 ng/ml human IL-6, 10 ng/ml human IL-23, 10 ng/ml human transforming growth factor beta (TGF-β); also included were beads coated with anti-CD2, anti-CD3, and anti-CD28 (T Cell Activation/Expansion kit from Miltenyi Biotec, Bergisch Gladbach, Germany). The cytokines were purchased from PeproTech (Rocky Hill, NJ, United States).

### Real-Time RT-PCR

RNA extraction was carried out using TRI Reagent (Molecular Research Center, Cincinnati, OH, United States). RNA was then reverse transcribed using the Maxima First Strand cDNA Synthesis Kit for RT-quantitative PCR (Thermo Fisher Scientific, Waltham, MA, United States). Real-time RT-PCR analysis was performed using SYBR Green I Master Mix on a LightCycler 480 from Roche (Basel, Switzerland). The reactions were run in a 384-well white plate at 95°C for 5 min, followed by 40 cycles of 95°C for 10 s, 60°C for 10 s, and 72°C for 20 s. The following primers were designed using Primer3 software (Rozen and Skaletsky, 2000): *G6PC*, 5′-TCCATACTGGTGGGTTTTGG-3′ (forward) and 5′-GAGGAAAATGAGCAGCAAGG-3′ (reverse); *NPAS2*, 5′-AGTCTGAGAAGAAGCGTCGG-3′ (forward) and 5′-TGTCACAGATTTCCGTTTGC-3′ (reverse); *IL-17A*, 5′-AAACAACGATGACTCCTGGG-3′ (forward) and 5′-CTTGTCCTCAGAATTTGGGC-3′ (reverse), as described previously (Ratajewski et al., 2012); and *IL-17F*, 5′-CTTTCTGAGTGAGGCGGC-3′ (forward) and 5′-TGGGAACGGAATTCATGG-3′ (reverse), as described previously (Ratajewski et al., 2016). The mRNA levels were normalized by the geometric mean of the levels of the housekeeping genes *HPRT1*, *HMBS*, *RPL13A*, as described by Vandesompele et al. (2002).

### Electrophoretic Mobility Shift Assay (EMSA)

Nuclear extracts were prepared from HepG2 cells using a Nuclear Extract Kit (Active Motif, Carlsbad, CA, United States). The DNA sequence of the RORE specific for RORγ was 5′-CGCGTGGTAAGTAGGTCACTCTC-3′ and was taken from the work of Medvedev et al. (1996) The probes were labeled with IRD-700 (infrared dye). The reaction was carried out on ice. DNA probes (10 fmol) were incubated with 1.25 μg of nuclear extract in a binding buffer containing 10 mM Tris-HCl (pH = 8.0), 50 mM KCl, 18.5 mM NaCl, 1 mM dithiothreitol (DTT), 0.1% IGEPAL, 5% glycerol, and 100 ng of salmon testis DNA. For the competition assay, 100-fold molar excesses of unlabeled oligonucleotides were added to the reaction mixture. For the supershift experiment, 200 ng of anti-ROR gamma antibody [162C2a] ab58670 from Abcam (Cambridge, Great Britain) was added. A 5% nondenaturing polyacrylamide gel was prerun for 20 min at 50 V. The samples were then added to the wells, and the gel was run at 130 V for 1.5 h at 4°C and then analyzed on an Odyssey (LiCor Biosciences, Lincoln, NE, United States) infrared fluorescence scanner.

### Chromatin Immunoprecipitation (ChIP)

Proteins in living cells were cross-linked with DNA by incubation in 1% formaldehyde-containing medium. Cells were harvested, lysed and then sonicated to form soluble chromatin using a VCX-130 sonicator (Sonics & Materials Inc., Newtown, CT, United States). Chromatin immunoprecipitation (ChIP) was performed using an EZ-Magna ChIP A/G Kit from EMD Millipore (Billerica, MA, United States) according to the manufacturer’s instructions. The following antibodies were used: normal mouse IgG (EMD Millipore) and anti-ROR gamma antibody [162C2a] ab58670 from Abcam. The relative enrichment of the *G6PC*, *NPAS2*, *IL17A*, and *IL17F* promoters was analyzed with real-time PCR using SYBR Green I Master Mix on a LightCycler 96 from Roche. The reactions were run in a 96-well white plate at 95°C for 10 min, followed by 40 cycles of 95°C for 20 s, 58°C for 20 s, and 72°C for 20 s. The primers used for the ChIP assay were as follows: 5′-CCAAAGTTAATCATTGGCCC-3′ (forward, *G6PC*) and 5′-TTGCCCCTGTTTTATATGCC-3′ (reverse, *G6PC*); 5′-CACTGGTGCAAAAGGAGAGG-3′ (forward, *NPAS2*) and 5′-ACTGCTGGGGAGGAATAACC-3′ (reverse, *NPAS2*) described previously (Karaś et al., 2018); 5′-GCAGCTCTGCTCAGCTTCTA-3′ (forward, *IL17A*) and 5′-GGGCTTTTCTCCTTCTGTGG-3′ (reverse, *IL17A*); 5′-CTCTGATTTGTGGGCAATGG-3′ (forward, *IL17F*) and 5′-CCGGAGTTACTGACGAATGC-3′ (reverse, *IL17F*). Soluble chromatin collected before immunoprecipitation was amplified as an input control. The relative PCR product enrichment was calculated using the dCt method, with the Ct obtained for the input control DNA as a reference value, as follows: 1000 × 2^−dCt^, where dCt = Ct sample – Ct input DNA.

### Analysis of IL-17 Production (ELISA)

The CD4+ fraction isolated from the buffy coats of healthy donors was differentiated under Th17-polarizing conditions in the presence of 100 nM digoxin for 5 days. Then, the culture supernatants were collected, and the IL-17 level was analyzed by ELISA using a Quantikine Human IL-17 Immunoassay Kit (R&D Systems) according to the manufacturer’s protocol. Microtiter plates were read on a Sunrise microplate reader (Tecan) at 405 nm.

### Preparation of Libraries, Sequencing, and RNA-Seq Data Analysis

Global changes in gene expression in human Th17 cells differentiated in the presence of 100 nM digoxin were analyzed by high-resolution RNA sequencing (RNA-seq). Libraries for sequencing were prepared using the reagents provided in the Illumina^®^ TruSeq^®^ RNA Sample Preparation Kit v2 (Sand Diego, CA, United States) according to the manufacturer’s instructions. The poly-A-containing mRNA molecules were purified from total RNA samples using magnetic beads with oligo-dT attached. Following purification, the mRNA was fragmented using divalent cations under elevated temperature. The cleaved RNA fragments were then reverse transcribed with random primers. The synthesis of the second strand of the cDNA was conducted using DNA Polymerase I and RNase H. Then, the cDNA fragments were subjected to an end repair process, the addition of a single “A” base, and ligation of the adapters. The final double-stranded cDNA library was purified and enriched with PCR. The libraries were then pooled together in the same molarity (10 nm) and were ready for clustering on a high-throughput flow cell. Sequencing of the samples was performed on a NextSeq 500 (Illumina) using 150 reads in pair-end mode.

The quality of the raw reads was checked using Fast QC software^1^. For filtering and trimming, bbduk2 v37.10 was used^2^. The obtained RNA-seq data that passed QC were mapped against the human transcriptome with an annotation file (GRCh38) obtained from ENSEMBL. For the mapping and depletion of human rRNA, we used Bowtie2 v2.2.6 (Langmead and Salzberg, 2012), and for the quantification, Salmon software v0.8.2 (Patro et al., 2017) was used. The Salmon quantification results were then used for downstream analysis to assess the differentially expressed genes. A three-way bioinformatic comparison between samples was conducted using the r packages DESeq v1.32 (Anders and Huber, 2010), DESeq2 v1.20.0 (Love et al., 2014), and edgeR v3.1 (Robinson et al., 2010) according to the estimated reads per kilobase per million mapped reads (RPKM).

### Gene Ontology Analysis

Gene ontology was performed using PANTHER software, version 11 (Mi et al., 2017).

### Docking Simulations

Molecular docking was performed as described in Karaś et al. (2018) with the following exceptions: as host structures, we chose the RORγ LBD structures with the following PDB IDs: 3B0W (Fujita-Sato et al., 2011), 3L0J (Jin et al., 2010), 5VB6, and 5VB7 (Li et al., 2017). The initial structures were retrieved from the Protein Data Bank (PDB) (Berman et al., 2000; Rose et al., 2017) as.pdb files. For the energy surface exploration, the Lamarckian Genetic Algorithm (Morris et al., 1998, 2009) was used, with the maximum number of energy evaluations set to the value 5.0 × 10^7^. The rest of the parameters of the optimization algorithm were left at their default values. For each pair of ligand-host systems, 20 runs were performed, resulting in the ranking of the most favorable orientations. To estimate the convergence of the algorithm, the docking simulation of the digoxin-3L0J system was carried out with an increased maximum number of energy evaluations of 1.0 × 10^8^. The energies and geometries of the most stable structures were fairly similar, and the free energy of binding was the same to within a few tenths of a kcal/mol.

### Statistics

Statistical significance was determined using one-way ANOVA followed by Tukey’s *post hoc* test, except for data obtained from different human donors, which were analyzed by the Wilcoxon Signed-Rank Test. A *p*-value of 0.05 or lower was considered statistically significant.

All relevant data is contained within the manuscript:

All datasets (GENERATED/ANALYZED) for this study are included in the manuscript and the Supplementary Materials.

## Results

### Digoxin at Noncytotoxic Concentrations Induces the Transactivating Function of RORγ in HepG2 Cells

**FIGURE 1:**
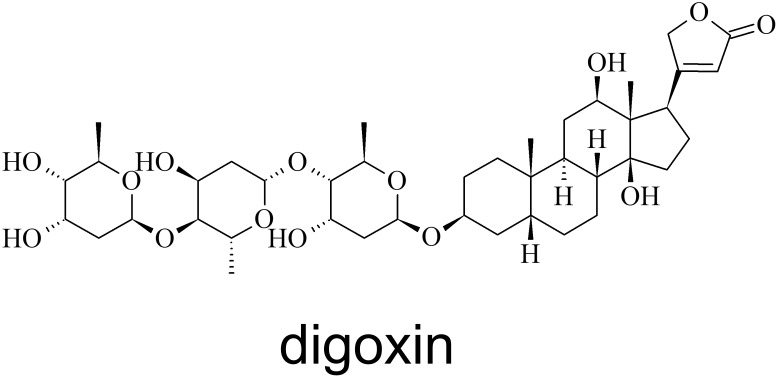
Structure of digoxin.

**FIGURE 2:**
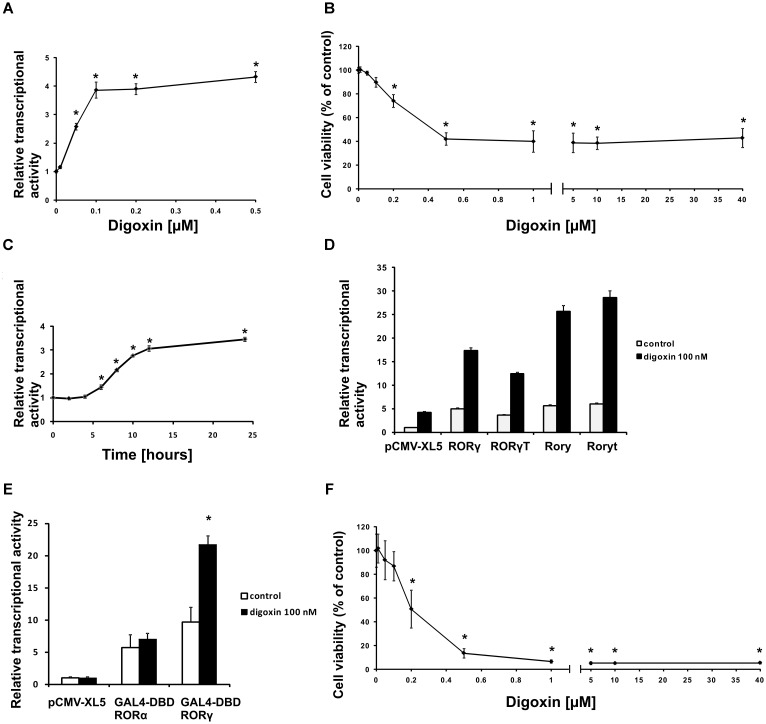
Digoxin activates RORγ-dependent transcription at subcytotoxic concentrations. **(A)** The effect of digoxin on RORγ-HepG2. Mean ± SD, *n* = 6. **(B)** The effect of digoxin on HepG2 cell viability. Mean ± SD, *n* = 6. **(C)** A time course of digoxin (100 nM) in the RORγ-HepG2 assay. Mean ± SD, *n* = 6. **(D)** Digoxin potentiates the effect of human RORγ and RORγT and mouse Rory and Roryt overexpression in the RORγ-HepG2 reporter cell line. Mean ± SD, *n* = 5. **(E)** Digoxin specifically activates the GAL4-DBD RORγ fusion protein in the HepG2-pGL4.35 reporter cell line. Mean ± SD, *n* = 3. **(F)** The effect of digoxin on Th17 cell viability. Mean ± SD, *n* = 4 (four different donors); ^∗^significantly different from control treatment at *p* < 0.05.

First, we used our previously validated (Karaś et al., 2018) RORγ-HepG2 reporter cell line to verify the ability of a wide spectrum of digoxin (Figure 1) concentrations to induce the RORγ-dependent expression of luciferase. As shown in Figure 2A, digoxin induced the reporter, which reached the highest activity at a digoxin concentration of 500 nM. When analyzing the viability of HepG2 cells after treatment with digoxin, we noticed that the highest noncytotoxic concentration of the compound was 100 nM, while 200 nM caused 25% cytotoxicity and 500 nM caused approximately 60% cytotoxicity (Figure 2B). The effect of digoxin on cell viability was also analyzed in Th17 cells, and we showed that 100 nM concentration is the highest non-cytotoxic concentration of digoxin, and all above significantly reduced cell viability. Concentrations above 500 nM resulted in a viability of 5% (Figure 2F). This is in general agreement with previous studies showing that digoxin concentrations above 100 nM are highly toxic to various human cells *in vitro* (Johansson et al., 2001). When analyzing the time course of the effects of digoxin on the RORγ-HepG2 reporter, we observed that significant induction occurred 8 h after treatment and continued to increase, reaching a maximum level at 24 h (Figure 2C). Next, we transfected human RORγ and RORγT and mouse Rory and Roryt cDNA into the RORγ-HepG2 reporter cell line to investigate the effects of digoxin on cells overexpressing these nuclear receptors and to determine if there are any species-specific effects on the reporter response. Digoxin enhanced the transcriptional function of both human RORγ/RORγT and mouse Rory/Roryt receptors (Figure 2D), however, induction of the reporter by the mouse receptors was stronger than that by the human homologs. To confirm that the observed effects of digoxin were mediated by the binding of digoxin to the LBD, we used a reporter system in which the ligand-binding domain of the RORγ receptor was fused to the GAL4 DNA-binding domain (Kumar et al., 2010). The transcriptional functions (binding to the GAL4 upstream activator sequence) of the resulting fusion protein depend on the constitutive activity of the LBD and on the binding of the potential ligand to the LBD. As shown in Figure 2E, the overexpression of GAL4-DBD RORγ in cells stably transfected with the pGL4.35[luc2P/9XGAL4UAS/Hygro] vector (HepG2-pGL4.35 cells) resulted in increased reporter activity, and treatment with 100 nM digoxin further increased the response of the reporter. We did not observe similar results for GAL4-DBD RORα, suggesting that, as previously shown by Huh et al. (2011), digoxin has affinity to RORγ but not to RORα.

**FIGURE 3:**
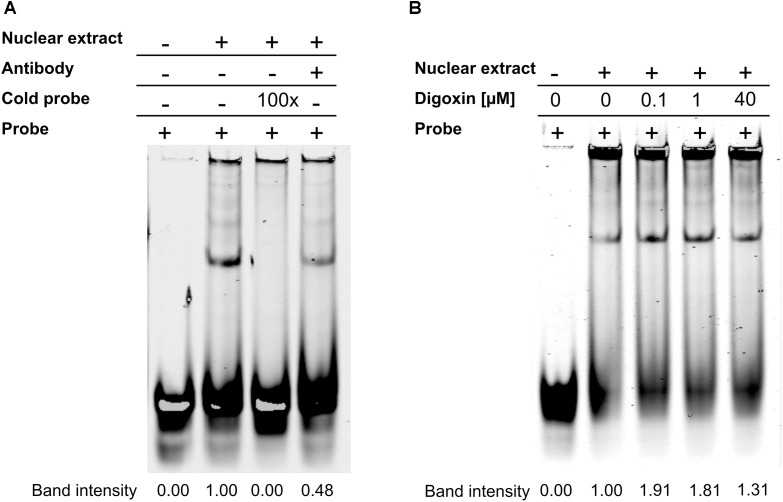
Results of the EMSA assay. **(A)** RORγ specifically binds to the considered RORE. **(B)** Digoxin increases binding of the protein extract to the considered RORE. Changing the brightness and contrast was applied equally across the entire images. Original scans are presented in Supplementary Materials.

In the next set of experiments, we tested whether digoxin was able to enhance RORγ binding to the RORE using EMSA. We used oligonucleotides containing a RORE specific for RORγ, as described previously by Medvedev et al. (1996), and nuclear extract isolated from HepG2 cells. We observed one band that was outcompeted by the 100-fold molar excess of unlabeled cold probe. The addition of the antibody against RORγ decreased the intensity of the band by approximately 50%, confirming that this protein binds to the probe (Figure 3A). The incubation of nuclear extract in the presence of increasing concentrations of digoxin caused an increase in the intensity of the band by 1.9- and 1.8-fold for 100 nM and 1 μM digoxin, respectively (Figure 3B).

**FIGURE 4:**
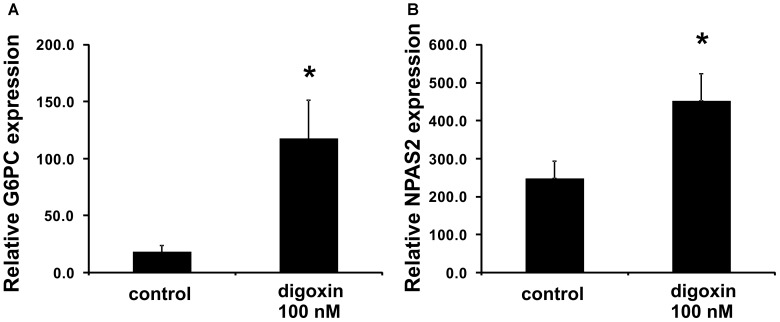
Effect of digoxin on the expression of RORγ-dependent genes in HepG2 cells. HepG2 cells were treated with digoxin (100 nM) for 24 h and then collected for RNA extraction. The expression of the **(A)** *G6PC* and **(B)** *NPAS2* genes was determined by real-time RT-PCR and normalized to the housekeeping genes *HPRT1*, *HMBS*, and *RPL13A*. Mean ± SD, *n* = 3, ^∗^statistically significant difference at *p* < 0.05 compared with control cells.

**FIGURE 5:**
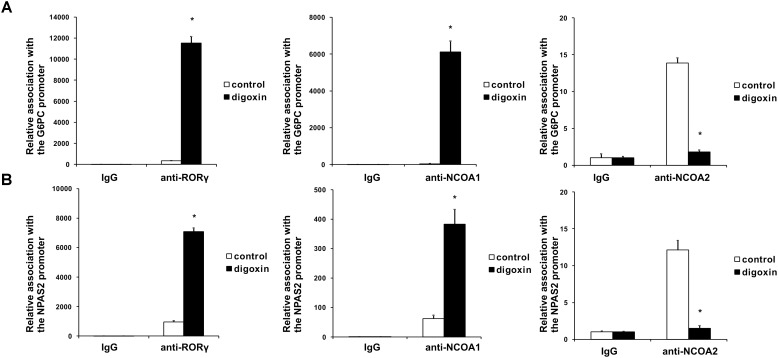
Analysis of the binding of RORγ, NCOA1 and NCOA2 *in vivo* to the promoter sequences of the *G6PC* **(A)** and *NPAS2* **(B)** genes in HepG2 cells after treatment with digoxin as evidenced by chromatin immunoprecipitation. Cells were treated with 100 nM digoxin for 8 h and then analyzed using chromatin immunoprecipitation. Mean ± SD, *n* = 3, ^∗^significantly different from the control treatment at *p* < 0.05.

When we analyzed the effect of digoxin on the expression of the RORγ-regulated genes *G6PC* and *NPAS2* (Wang et al., 2010b; Takeda et al., 2011, 2014), we observed their increased expression (Figure 4). This was accompanied by increases in *G6PC* and *NPAS2* promoter occupancy by RORγ, as evaluated using the ChIP technique (Figure 5). It is known that to show full transactivation potential, RORγ needs to interact with coactivators (Kurebayashi et al., 2004; Wang et al., 2010a; Kojima et al., 2015; Takahashi et al., 2017), which is why we next performed ChIP analysis using anti-NCOA1 (SRC-1) and anti-NCOA2 (SRC-2) antibodies to determine the status of these coactivators on the *G6PC* and *NPAS2* promoters. Similar to the results for RORγ, digoxin treatment increased *G6PC* and *NPAS2* promoter occupancy by NCOA1 and decreased *G6PC* and *NPAS2* promoter occupancy by NCOA2 (Figure 5).

### Digoxin Activates RORγT in Human Th17 Cells

**FIGURE 6:**
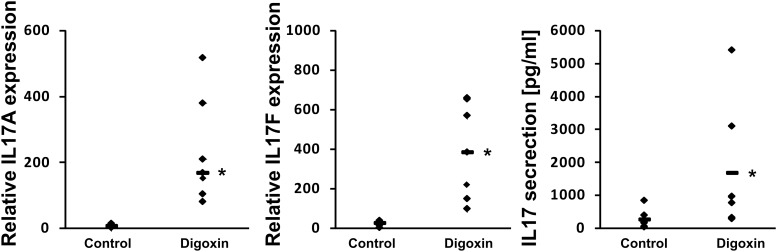
Effect of digoxin on the expression of RORγT-dependent genes in human Th17 cells. Human naive CD4+ cells were treated with 100 nM digoxin and then cultured under Th17-polarizing conditions for 5 days. After that time, cells were collected for RNA extraction and supernatants were collected for IL-17 secretion analysis. The expression of the *IL17A* and *IL17F* genes was determined by real-time RT-PCR and normalized to the housekeeping genes *HPRT1*, *HMBS*, and *RPL13A*. Analysis of IL-17 production was performed using a Quantikine Human IL-17 Immunoassay kit (R&D Systems). An asterisk indicates a statistically significant difference at *p* < 0.05 compared with control cells. The data are presented as statistical dot plots with the median value (bars) from seven independent experiments performed using cultures originating from seven different donors (*n* = 7).

**FIGURE 7:**
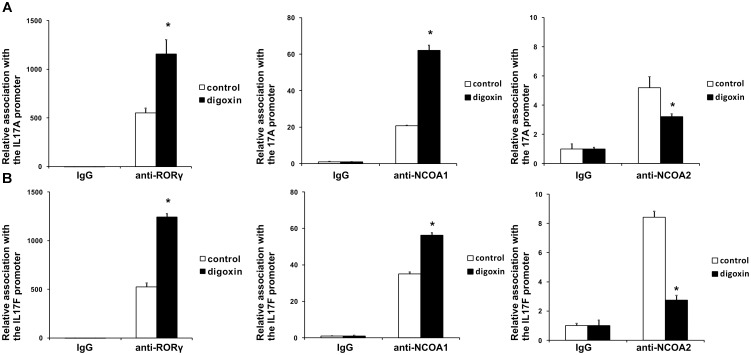
Analysis of the binding of RORγT, NCOA1 and NCOA2 *in vivo* to the promoter sequences of the *IL17A* **(A)** and *IL17F* **(B)** genes in Th17 cells after treatment with digoxin as evidenced by chromatin immunoprecipitation. Human naive CD4+ cells were treated with 100 nM digoxin, cultured under Th17-polarizing conditions for 5 days and then analyzed using chromatin immunoprecipitation. Mean ± SD, *n* = 3 (from three different donors), ^∗^significantly different from the control treatment at *p* < 0.05.

The shorter isoform of the RORC gene, RORγT, in humans is almost exclusively expressed in Th17 lymphocytes, where it regulates Th17-specific cytokines (IL17A and IL17F) and is considered a signature transcription factor of these cells (Crome et al., 2009). To determine whether digoxin is able to influence the differentiation of Th17 lymphocytes, we cultured naive CD4+ cells under Th17-polarizing conditions in the presence of 100 nM digoxin. We observed that treatment with digoxin substantially increased the mRNA levels of *IL17A* and *IL17F* and, as a consequence, increased the concentration of IL17 protein in cell culture supernatants (Figure 6). Upon analyzing the occupancy status of the RORγT, NCOA1 and NCOA2 proteins on the *IL17A* and *IL17F* promoters by ChIP, we observed a similar pattern as previously observed in HepG2 cells: digoxin increased occupancy by RORγT and NCOA1 (Figure 7), while NCOA2 binding was decreased. To provide further insight into changes in gene expression upon digoxin treatment, we analyzed the transcriptomes of Th17 cells (originating from four different donors) cultured in the presence of 100 nM digoxin. Detailed analysis with three different programs (Deseq, Deseq2 and edgeR) allowed us to identify 1693 transcripts whose expression changed upon digoxin treatment (Supplementary Table 2). Gene ontology analysis (with PANTHER software) revealed that some of the GO terms related to the biology of T-cells were overrepresented (Table 1). Among the genes that were induced were cell membrane proteins expressed by Th17 cells (Acosta-Rodriguez et al., 2007; Annunziato et al., 2007), including *CCR4*, *CCR6*, and *IL23R*; Th17-specific interleukins, including *IL17A*, *IL17F*, *IL21*, and *IL22* (Liang et al., 2006; Veldhoen et al., 2006; Fouser et al., 2008; Vogelzang et al., 2008); and Th17 differentiation regulators, including *STAT3*, *AhR*, and *VDR* (Nishihara et al., 2007; Yang et al., 2007; Kimura et al., 2008; Chang et al., 2010; Joshi et al., 2011; Ciofani et al., 2012; Table 2).

**Table 1:** Gene ontology (biological process) term results from the PANTHER overrepresentation test for significantly differentially expressed (DE) genes after digoxin treatment in Th17 cells.

GOID	GO biological process term	Number of genes	*p*-value
GO:0001775	Cell activation	115	1.93 × 10^−4^
GO:0045321	Leukocyte activation	97	1.18 × 10^−3^
GO:0042102	Positive regulation of T cell proliferation	20	4.15 × 10^−4^
GO:0030155	Regulation of cell adhesion	75	6.44 × 10^−4^
GO:0042129	Regulation of T cell proliferation	28	1.26 × 10^−4^
GO:0050670	Regulation of lymphocyte proliferation	39	2.81 × 10^−6^
GO:0032944	Regulation of mononuclear cell proliferation	40	1.42 × 10^−6^
GO:0070663	Regulation of leukocyte proliferation	41	1.10 × 10^−6^
GO:0050671	Positive regulation of lymphocyte proliferation	27	4.46 × 10^−5^
GO:0032946	Positive regulation of mononuclear cell proliferation	28	2.25 × 10^−5^
GO:0070665	Positive regulation of leukocyte proliferation	28	2.89 × 10^−5^

*GOID: gene ontology term identifier; number of genes: the number of genes mapped to specific GO term; p-value: the expected value calculated by Fisher’s exact test with false discovery rate multiple test correction (p < 0.05) for the overrepresentation of selected DE genes in the GO category.*

**Table 2:** Literature-validated Th17 signature genes (from Ciofani et al., 2012) selected from the significantly differentially expressed (DE) genes after digoxin treatment in Th17 cells determined using RNA-seq.

Gene name	Full name	Fold change
*AHR*	Aryl hydrocarbon receptor	3.140
*CCR4*	C-C motif chemokine receptor 4	2.051
*CCR6*	C-C motif chemokine receptor 6	2.842
*CSF2*	Colony stimulating factor 2	53.399
*DEF6*	DEF6, guanine nucleotide exchange factor	0.519
*EBI3*	Epstein-Barr virus induced 3	4.682
*FOSL2*	FOS Like 2, AP-1 transcription factor Subunit	2.302
*IL10*	Interleukin 10	3.523
*IL17A*	Interleukin 17A	35.174
*IL17F*	Interleukin 17F	13.879
*IL21*	Interleukin 21	5.579
*IL22*	Interleukin 22	3.796
*IL23R*	Interleukin 23 receptor	6.066
*KSR1*	Kinase suppressor of ras 1	2.428
*NDFIP1*	Nedd4 family interacting protein 1	2.086
*NFKB1*	Nuclear factor kappa B subunit 1	2.264
*PTGER4*	Prostaglandin E receptor 4	2.974
*RBPJ*	Recombination signal binding protein for immunoglobulin kappa J region	1.793
*SIGIRR*	Single Ig and TIR domain containing	0.599
*STAT3*	Signal transducer and activator of transcription 3	1.683
*TGFBR2*	Transforming growth factor beta receptor 2	0.480
*TNFRSF8*	TNF receptor superfamily member 8	7.095
*TXK*	TXK tyrosine kinase	0.320
*VDR*	Vitamin D receptor	2.705

*Fold change: the difference in gene expression levels between digoxin-treated and untreated Th17 cells was counted as a mean from the fold change of DESeq, DESeq2 and edgeR analyzes.*

### Final Docked Poses Analysis

In the current study, we considered 5 host domains of RORγ. All of these were considered in the context of the binding of the digoxin system through the molecular docking approach. The key technical issues of the overall computational protocol have been briefly described in the experimental section.

**FIGURE 8:**
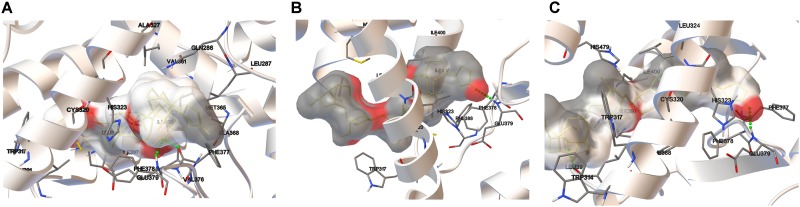
Docking results for digoxin using the crystal structures of the RORγ LBD: 3L0J **(A)**, 3B0W A **(B)**, and 3B0W B **(C)**. Amino acid residues are shown where relatively close ligand-receptor contact occurs. The ligand is surrounded by the electrostatic potential surface (based on the point charges). Hydrogen bonds are represented as green dotted lines.

The estimated free energies of binding are presented in Table 3, and their graphical representation is presented in Figure 8 and Supplementary Figure 1. The host domain is represented as a white ribbon, and only the residues that are in close contact with the ligand system are explicitly shown. The interactions with these explicitly named residues provide contributions to overall binding. Due to the complicated nature of intermolecular interactions, it is a difficult task to precisely decompose the interaction into the contributions coming from the separate residues. On the other hand, one can safely assume that a significant component of the interaction energy is provided by the hydrogen bonds. Each considered host domain constitutes one or more hydrogen bonds with the digoxin molecule.

**Table 3:** The lowest and mean free energy of binding are presented for each considered host domain.

Host	Estimated free energy of binding (kcal/mol)		Number of items in cluster
domain		
	Lowest	Mean	
3L0J	−13.85	−12.56	3
3B0W_A	−11.48	−11.06	3
3B0W_B	−11.93	−11.58	3
5VB6	−10.11	−8.81	4
5VB7	−9.99	−9.99	1

*The number of members of the cluster is also provided.*

The most significant docking score was obtained in the case of the 3L0J RORγ domain (see Figure 8A). The most significant interaction between the ligand and host system is formed by two hydrogen bonds where the phenylalanine (PHE377) residue interacts with the hydroxyl group and the same hydroxyl group forms a hydrogen bond with glutamic acid (GLU379). The significant docking score of the 3B0W domains (A and B) (Figures 8B,C) is due to the strong hydrogen bond formed between the amine group of glutamic acid (GLU379) and the carbonyl group of the terminal five-member ring of digoxin. The remaining two domains (with the SRC-2 peptide) are characterized by lower binding energies (Table 3). In both, we observed different orientations of digoxin within the binding pocket. The carbonyl group of the terminal five-member ring of digoxin is close to the histidine (HIS479) residue. In the case of the 5VB6 domain, the digoxin molecule forms a hydrogen bond between the imidazole part of HIS479 and the carbonyl group of the digoxin five-member ring (Supplementary Figure 1A). In the case of the 5VB7 domain, the HIS479 residue is still close to the carbonyl group, but explicit hydrogen bonds are formed by glutamic acid (GLU326) and glutamine (GLN286) interacting with the same hydroxyl group of digoxin (Supplementary Figure 1B).

## Discussion

Cardiac glycosides have a long history of use, especially in folk medicine (Kelly, 1990). In modern times, they were a part of therapy to treat heart ailments, especially atrial fibrillation and congestive heart failure (Kelly, 1990; Patel, 2016), but due to the risk of overdose and high toxicity (Fozzard and Sheets, 1985), they have been replaced by synthetic drugs. The most broadly employed cardiac glycoside was digoxin (others were digitoxin, ouabain and lanatoside C) (Doherty et al., 1978), which exerts its biological effect through inhibition of the Na^+^/K^+^-ATPase (Katz, 1985). Digoxin was also one of the first identified inverse agonists of RORγT (Huh et al., 2011), however, the compound exerts its inhibitory activity against mouse Roryt and human RORγT at very high concentrations [10 and 40 μM, respectively (Fujita-Sato et al., 2011; Huh et al., 2011)], which precludes its potential clinical application. Previously, in a screen of 2400 compounds, we identified several cardenolides that were able to activate RORγ and RORγT-dependent transcription in human cells at low noncytotoxic concentrations (Karaś et al., 2018). This prompted us to reinvestigate the effects of digoxin, which structurally is very similar to these compounds (Figure 1). We determined that the range of concentrations of digoxin not causing detectable cytotoxicity is below 100 nM. Using two cell reporter systems, we found that digoxin at such nontoxic concentrations is able to induce a RORγ-dependent reporter in intact cells (Figure 2) and to increase the binding of the RORγ protein to the RORE in *in vitro* assays (Figure 3). Furthermore, digoxin mediated upregulation of the expression of the *G6PC* and *NPAS2* genes (Figure 4) and occupancy of the RORγ protein and NCOA1 coactivator on their promoters in HepG2 cells (Figure 5). Digoxin also mediated increased binding of RORγT on the *IL17A* and *IL17F* promoters and higher expression of these genes in differentiating Th17 cells (Figures 6, 7). To better characterize the effects of digoxin on the differentiation of Th17 cells, we analyzed the transcriptomes of Th17 cells differentiating in the presence of 100 nM of the compound. The results revealed that digoxin increased the expression of genes coding for phenotypic markers of the Th17 cell lineage (Table 2).

Our results are in striking contrast to those of a previously published work by Huh et al. (2011), in which authors did not observe a stimulatory effect of low concentrations of digoxin on the RORγ reporter in the Drosophila S2 cell line. One possible explanation for these differences is that the transactivating function of RORγ depends on the binding of coactivators. It is quite probable that insect cells not expressing the human protein repertoire do not provide the optimal model for the observation of the stimulatory effect of digoxin on RORγ activity. This hypothesis is supported by the observation of a digoxin-mediated increase in the binding of the NCOA1 coactivator and a decrease in the binding of the NCOA2 protein in loci containing ROREs (Figures 5, 7), suggesting the involvement of coactivators in the process of digoxin-mediated RORγ activity. The hypothesis that digoxin acts as a RORγ agonist is also supported by the molecular docking analysis, in which the binding of digoxin to the LBD of RORγ in different conformations (agonistic and inverse agonistic) revealed that the most significant docking scores were obtained for the agonistic conformation (Table 3 and Figure 8A). Interestingly, docking analysis with LBDs containing the SRC-2 peptide (5VB6 and 5VB7) resulted in lower docking scores (Table 3), suggesting that the presence of the SRC-2 (NCOA2) coactivator in the LBD-digoxin complex is undesirable, which is in full agreement with our experimental data obtained by ChIP assays (Figures 5, 7). There are several reports of compounds, including methylhonokiol (Gertsch and Anavi-Goffer, 2012) and propranolol (Baker et al., 2003), that act as both agonists and inverse agonists. The difference between these compounds and digoxin is that while the compounds exhibit both inverse agonist and agonist activity at similar ranges of concentrations, digoxin agonistic activity was observed at concentrations 400 times lower than those reported to mediate inverse agonist action in humans. Considering the differences in experimental systems employed in both studies, it is still conceivable that digoxin is capable of exhibiting both agonist and inverse agonist activity.

The therapeutic ranges of digoxin do not exceed 1.2 ng/ml (approximately 1.5 nM) due to possible side effects (Goldberger and Goldberger, 2012). The concentrations that we tested in the present study were 30–60 times higher and were comparable to those detected in massive digoxin overdoses (Smith and Willerson, 1971; Citrin et al., 1972) associated with significant adverse effects, thus ruling out the use of digoxin as a RORγ agonist in patients. On the other hand, low concentrations of cardenolides (digoxin and ouabain) were found to be synthesized in mammalian cells as endogenous digitalis-like factors (EDLF) regulating the ion gradient between cells and the extracellular fluid (Hamlyn et al., 1991; Blaustein, 2014; Buckalew, 2015). These observations suggest that, similar to the case for hormones, the concentrations of cardenolides necessary to observe their bioactivity in *in vitro* studies do not reflect physiological situations (Vandenberg et al., 2012).

Th17 lymphocytes have been implicated in cancer, and their role depends on several factors (e.g., the type of cancer and the cytokine milieu) (Bailey et al., 2014). These lymphocytes are promising targets for adoptive cell therapy (ACT) (Muranski et al., 2008; Canderan and Dellabona, 2010) since they have better properties for retaining antitumor activity than Th1 and CD8+T cells (Bowers et al., 2017). The use of agonists or activators of RORγ/RORγT, such as digoxin, that are capable of promoting Th17 signaling in ACT seems to be a potentially attractive translation of our findings into clinical application, especially considering the lower risk of toxic side effects in *ex vivo* treatment with this compound.

## Author Contributions

MR designed and supervised the study. KK, AS, MS-K, AW-D, and MR performed the experiments. RB performed the molecular docking analysis. DS, JD, RB, and MR analyzed the data. JD, RB, and MR wrote the manuscript.

## Conflict of Interest Statement

The authors declare that the research was conducted in the absence of any commercial or financial relationships that could be construed as a potential conflict of interest.

## Footnotes

1 http://www.bioinformatics.babraham.ac.uk/projects/fastqc

2 https://jgi.doe.gov/data-and-tools/bbtools/bb-tools-user-guide/bbduk-guide/

## Abbreviations

AhR: aryl hydrocarbon receptor
CCR4: C-C motif chemokine receptor 4
CCR6: C-C motif chemokine receptor 6
ChIP: chromatin immunoprecipitation
EMSA: electrophoretic mobility shift assay
G6PC: glucose-6-phosphatase catalytic subunit
HMBS: hydroxymethylbilane synthase
HPRT1: hypoxanthine phosphoribosyltransferase 1
IL23R: interleukin 23 receptor
NCOA1: nuclear receptor coactivator 1
NCOA2: nuclear receptor coactivator 2
NPAS2: neuronal PAS domain protein 2
NR: nuclear receptor
RORC: RAR related orphan receptor C
RORγ: nuclear receptor ROR-gamma isoform 1
RORγT: nuclear receptor ROR-gamma isoform 2
RORα: RAR related orphan receptor A
RORE: ROR response element
RPL13A: ribosomal protein L13a
STAT3: signal transducer and activator of transcription 3
Th17: T helper 17 cell
VDR: vitamin D receptor.
