# Supplementary material for: Digoxin, an Overlooked Agonist of RORγ/RORγT
Source: Front Pharmacol. 2019 Jan 7;9:1460. doi: 10.3389/fphar.2018.01460 (PMC6330298; doi:10.3389/fphar.2018.01460)
Supplement: Supplementary file 1 [file Table_1.doc]

Supporting information

Digoxin, an overlooked agonist of RORγ/RORγT

Kaja Karaś, Anna Sałkowska, Marta Sobalska-Kwapis, Aurelia Walczak-Drzewiecka, Dominik Strapagiel, Jarosław Dastych, Rafał A. Bachorz and Marcin Ratajewski

Table of contents

1. Supplementary Figure 1…………………………………………………………S3
2. Supplementary Figure 2…………………………………………………………S4
3. Supplementary Figure 3…………………………………………………………S4
4. Supplementary Table 2 (uploaded as separate xls.file)……………………...S5


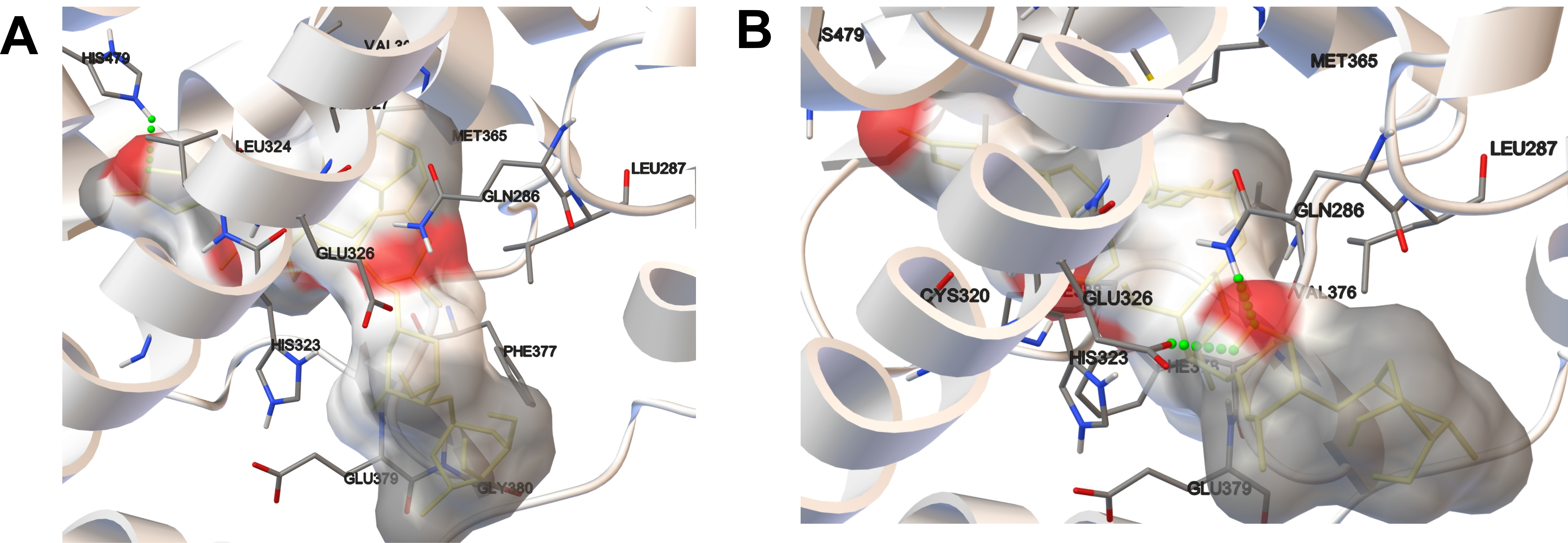


Supplementary Figure 1. Docking results for digoxin using the crystal structures of the RORγ LBD: 5VB6 (A) and 5VB7 (B). Amino acid residues are shown where relatively close ligand-receptor contact occurs. The ligand is surrounded by the electrostatic potential surface (based on the point charges). Hydrogen bonds are represented as green dotted lines.


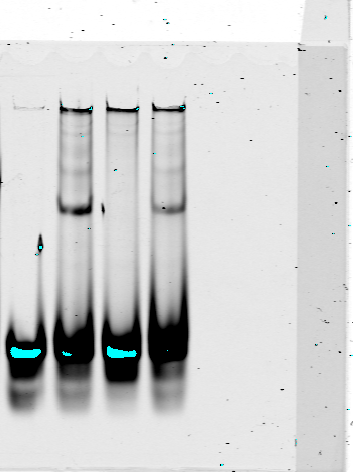


Supplementary Figure 2. Original scan of the EMSA gel presented in the Figure 3 (left panel).


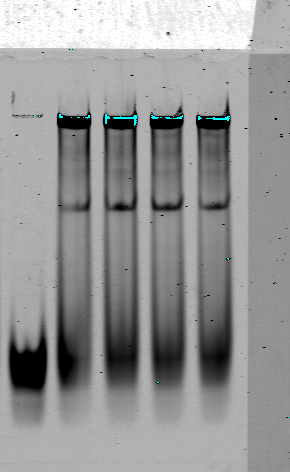


Supplementary Figure 3. Original scan of the EMSA gel presented in the Figure 3 (right panel).

Supplementary Table 2. A complete list of genes that changed expression upon digoxin treatment in Th17 cells determined using RNA-seq. Human naive CD4+ cells were treated with 100 nM digoxin and then cultured under Th17-polarizing conditions for 5 days. After that time, cells were collected for RNA extraction and analysed using RNA-seq from four independent experiments performed using cultures originating from four different donors (n=4). Results are uploaded as separate xls.file.
